# Late breast recurrence of plasmablastic lymphoma with apparent partial spontaneous regression

**DOI:** 10.1016/j.radcr.2026.09.015

**Published:** 2026-09-29

**Authors:** Maryam Alheddi, Najla Alomani, Amina Mohammed, Rabab Alkhayyat, Ahmed Alsharakhat, Raja Eid, Salwa Ali

**Affiliations:** a Radiology department, Salmaniya Medical complex, Manama, Bahrain; b Pathology department, Salmaniya Medical Complex, Manama, Bahrain; c Surgery department, Salmaniya Medical Complex, Manama, Bahrain; d Radiology Department, King Hamad University Hospital, Muharraq, Bahrain

**Keywords:** Lymphoma, Breast, Mammography, Spontaneous regression

## Abstract

Plasmablastic lymphoma (PBL) is an uncommon, highly aggressive lymphoma with a predilection for extranodal involvement. Secondary breast involvement is exceedingly rare and can closely resemble primary breast malignancy on imaging.

We present a 60-year-old woman with a remote history of sinonasal PBL in long-term remission who developed a rapidly enlarging right breast lump nearly two decades later. Initial breast imaging identified a highly suspicious lesion suggestive of malignancy, and ultrasound-guided core needle biopsy confirmed disease recurrence. Follow-up imaging performed one month later unexpectedly showed apparent partial spontaneous regression of the lesion. However, subsequent metabolic imaging identified widespread disease.

This case highlights the diagnostic complexity of breast involvement by PBL and the importance of accurate histopathological subtyping for prognostic assessment. It also emphasizes the value of integrating functional and morphologic imaging for accurate disease evaluation and optimal management.

## Introduction

Plasmablastic lymphoma (PBL) is a rare and aggressive subtype of diffuse large B-cell lymphoma (DLBCL) first described in HIV-positive individuals. However, subsequent literature has reported its occurrence in Epstein–Barr virus (EBV)-positive patients, immunocompromised patients, and even immunocompetent patients [1,2]. While the oral cavity remains the most common site of involvement, PBL has also been identified in other locations, including the pharynx, stomach, skin, lungs, pericardium, anorectal region, and various head and neck sites, such as the nasal, paranasal, and submandibular regions [3].

Lymphomas constitute an uncommon subtype of breast malignancies, accounting for only 0.04% to 1% of all breast cancer cases, while breast involvement is observed in approximately 1.7% of all extranodal lymphomas [4]. These tumors typically exhibit non-specific clinical and radiological manifestations. Therefore, histopathological assessment, immunohistochemical and genetic analysis are essential for establishing a definitive diagnosis [[5], [6], [7]].

An aggressive clinical course, poor prognosis, and a high risk of relapse characterize PBL. According to the World Health Organization (WHO), intensive multi-agent chemotherapy regimens such as dose-adjusted EPOCH, CODOX-M/IVAC, or Hyper-CVAD with central nervous system (CNS) prophylaxis are recommended [8]. Although rare, a few documented cases have reported spontaneous regression of PBL. The underlying mechanisms remain poorly understood; however, some hypotheses suggest that various factors may trigger immune system activation, leading to antitumor responses [9].

We present a 60-year-old immunocompetent woman with a history of treated sinonasal PBL who developed a late recurrence in the breast, which subsequently demonstrated apparent partial spontaneous regression on serial breast imaging.

## Case presentation

A 60-year-old immunocompetent woman initially presented 19 years earlier with a left maxillary sinus mass detected on CT images. She underwent surgical excision of the lesion, and histopathological examination confirmed PBL. Subsequent staging investigations demonstrated disease confined to the maxillary sinus with no evidence of regional or distant involvement, so adjuvant radiotherapy was administered. The patient subsequently underwent serial surveillance imaging for 10 years, which demonstrated sustained resolution of the previously identified imaging abnormalities, with no evidence of local recurrence or distant disease during follow-up.

She presented to our hospital with a 2-month history of a palpable lump in the right breast. On clinical examination, a semi-mobile mass was palpated in the central outer right breast, measuring around 7 cm in its largest dimension. Mammogram revealed an irregular dense mass with indistinct margins in the retro-areolar region of the right breast with increased surrounding trabeculation, and no associated suspicious calcifications. This was accompanied by ipsilateral prominent axillary lymph nodes (Fig. 1). The lesion demonstrated moderately suspicious imaging features, corresponding to a BI-RADS 4B assessment. A complementary breast ultrasound revealed a large, heterogeneous, predominantly hypoechoic, irregular solid mass with indistinct margins, measuring approximately 7.0 × 4.0 × 5.9 cm (Fig. 2). The overall findings were classified as BI-RADS 5, indicating a high suspicion for malignancy. Additionally, a few prominent ipsilateral axillary lymph nodes with preserved fatty hilum were noted.

**Fig. 1:**
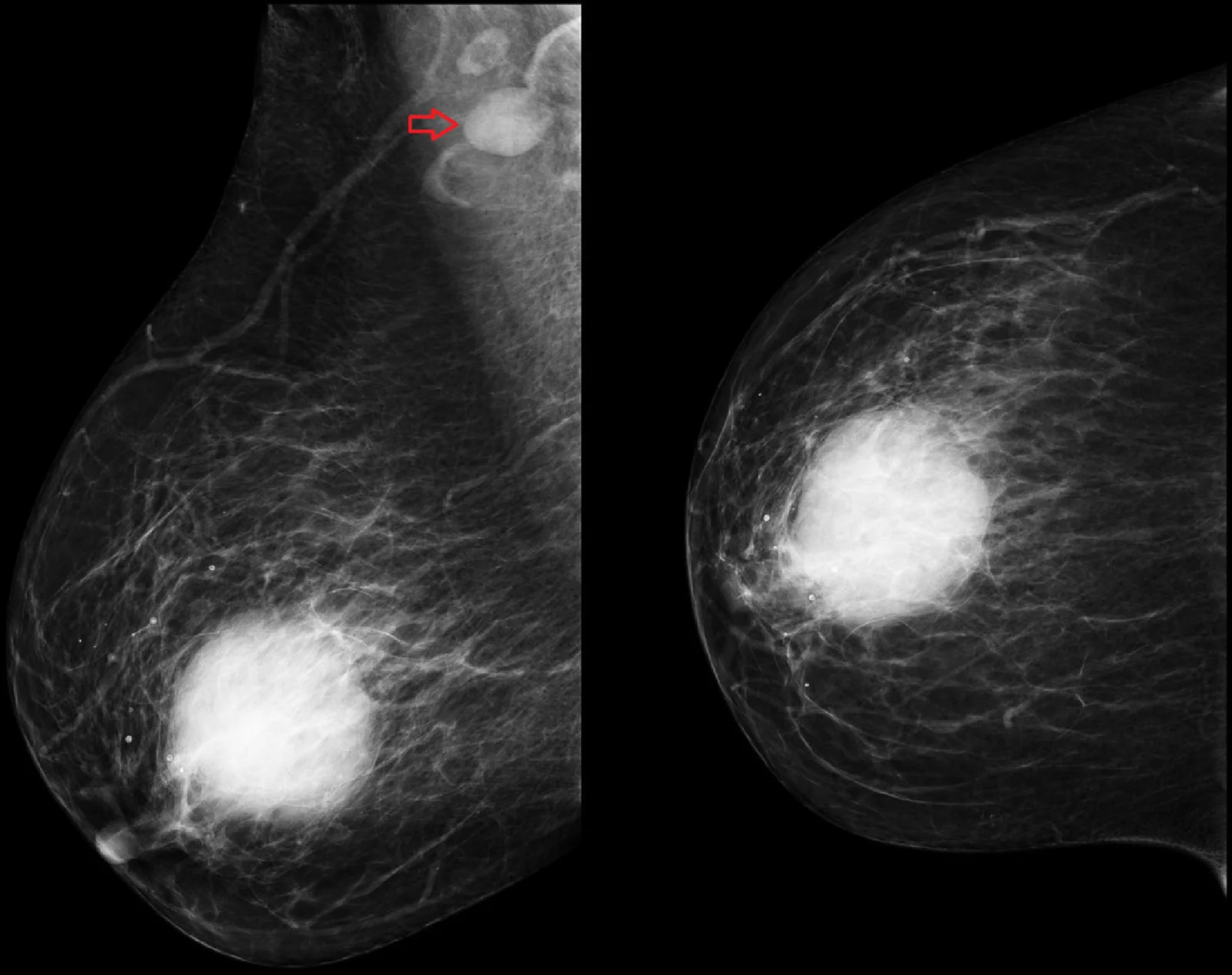
MLO and CC mammographic views of the right breast show an irregular dense mass with indistinct margins in the retroareolar region with increased surrounding trabeculation and mild stromal distortion. No associated suspicious calcifications. This is associated with prominent axillary lymphadenopathy (Red arrow). Abbreviations: CC, craniocaudal; MLO, mediolateral oblique.

**Fig. 2:**
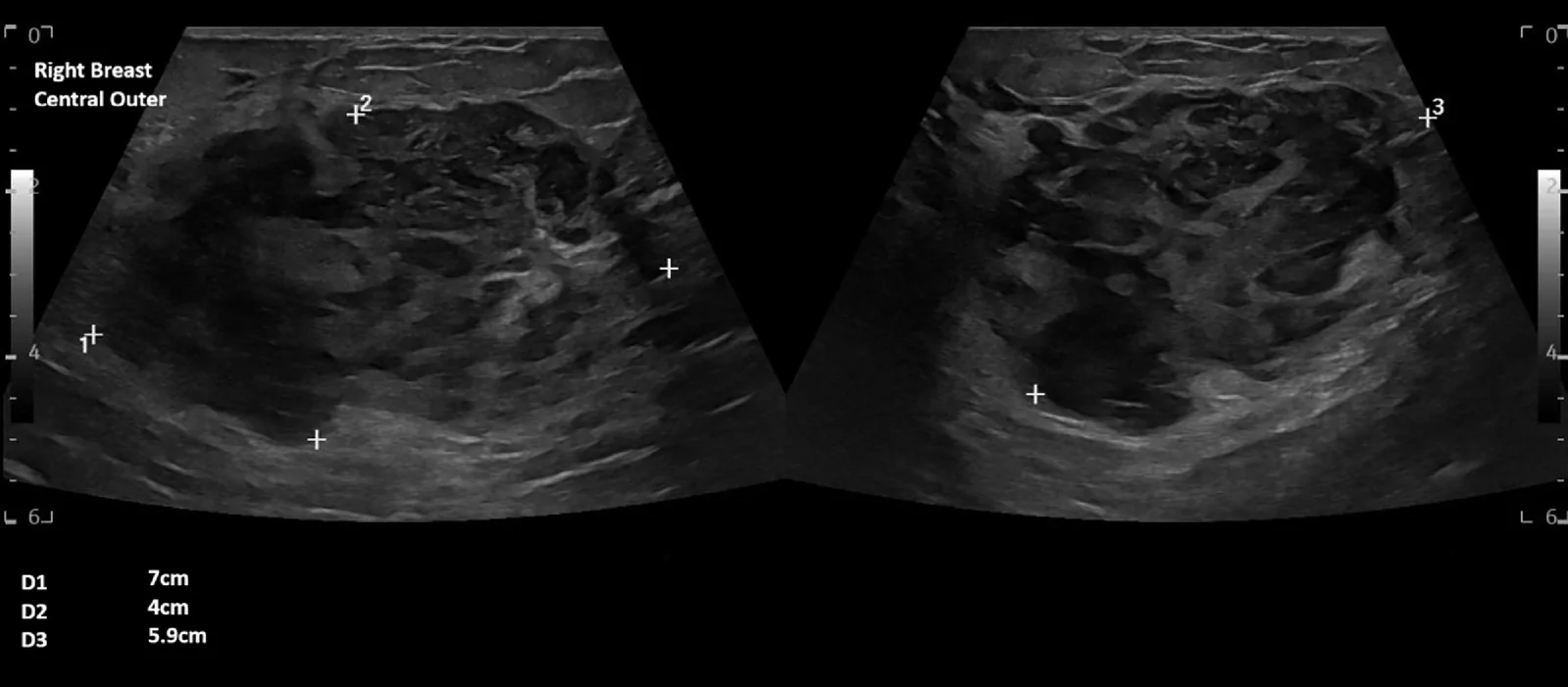
Ultrasound of the right breast shows a 7.0 × 4.0 × 5.9 cm irregular, heterogeneous, predominantly hypoechoic mass with indistinct margins located at the retro-areolar region extending to the outer quadrants (9 o'clock). Correlated with mammography, the findings were classified as BI-RADS 5 according to the American College of Radiology (ACR) assessment.

Ultrasound-guided core needle biopsies of the mass and axillary lymph node were performed. Histopathological examination showed diffuse infiltration of large, atypical lymphoid cells with plasmablastic morphology (Fig. 3). Immunohistochemical staining was positive for MUM1 and CD138, and negative for CD20, CD3, estrogen receptor (ER), progesterone receptor (PR), HER2, E-cadherin, GATA-3, and SOX10. Ki67 proliferation index is approximately 100%, and MYC is approximately 40%, consistent with PBL. No evidence of metastatic involvement was identified in the sampled axillary lymph node.

**Fig. 3:**
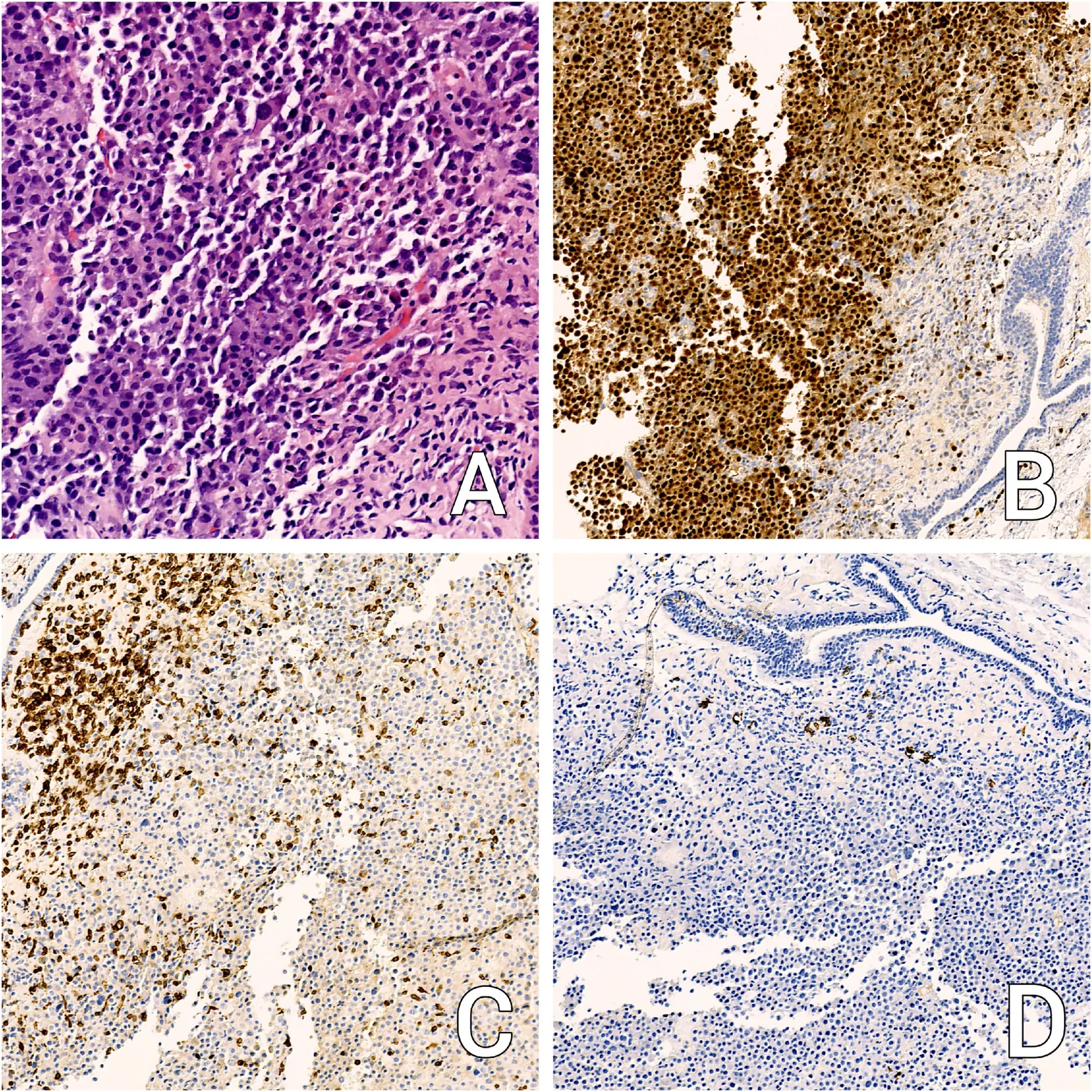
Core needle biopsy of the right breast mass. Hematoxylin and eosin staining (Image A, × 20 magnification) demonstrates breast tissue diffusely infiltrated by sheets of large atypical lymphoid cells with plasmablastic morphology. Immunohistochemical studies show that these atypical cells are positive for MUM1 (Image B) and negative for CD3 (Image C) and CD20 (Image D), consistent with plasmablastic lymphoma. These histopathological findings are concordant with the radiological features.

Unexpectedly, upon reassessment at the one-month follow-up visit in the oncological surgical clinic, the mass was no longer palpable on physical examination. Findings were confirmed independently by two clinicians, prompting repeat imaging. On follow-up mammography, no discrete residual mass was identified at the site of the previously documented lesion, although mild residual architectural distortion was noted. The current mammographic examination did not visualize the previously described axillary lymph nodes. Targeted breast ultrasound demonstrated no discrete residual mass or focal sonographic abnormality at the site of the previously identified lesion. Overall, the findings showed apparent radiologic resolution of the previously documented mass, with mild residual architectural distortion at the corresponding site (Fig. 4). Two radiologists, including a breast imaging consultant and a board-certified radiologist, independently reviewed and confirmed the imaging findings.

**Fig. 4:**
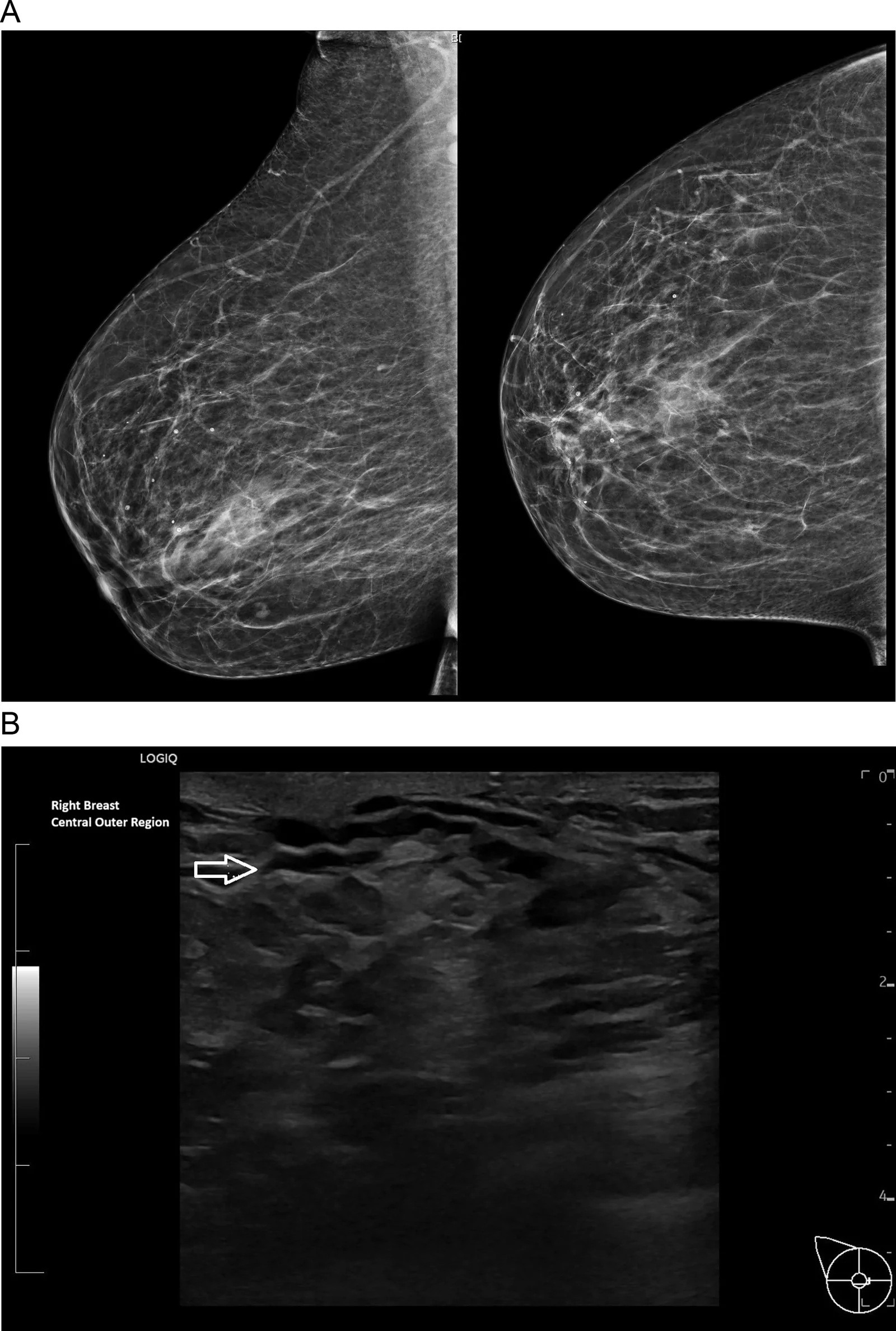
One-month follow-up mammography (Image A) and Ultrasound (Image B) show no discrete residual mass was identified at the site of the previously documented lesion, with mild residual architectural distortion noted (Image B, arrow). Overall, the findings showed apparent radiologic resolution of the previously documented mass, with mild residual architectural distortion at the corresponding site.

The patient was referred to the oncological hematological clinic for further assessment. Serological laboratory investigations were negative for HIV and EBV. Bone marrow biopsy showed preserved marrow architecture with no signs of infiltration. Baseline pre-treatment 18F-FDG PET/CT demonstrated a small FDG-avid, ill-defined right breast retro-areolar thickening measuring 4 cm (SUVmax ∼ 3) (Fig. 5, Image A&B, Black arrow), as well as a posterior mediastinal aorto-esophageal nodal lesion measuring 1.4 × 2.3 cm (SUVmax ∼ 7.8) (Fig. 5, Image A & C, Blue arrow), and bilateral pulmonary nodules with the most FDG-avid lesion in the right mid-lung zone para-cardiac region measuring 1.9 cm (SUVmax ∼ 6.8) (Fig. 5, Image A & D, star). A late systemic relapse of PBL was diagnosed, and chemotherapy with V-EPOCH was initiated. Follow-up 18F-FDG PET/CT performed two months later, after two cycles of V-EPOCH, demonstrated complete metabolic resolution of the breast lesion and posterior mediastinal aorto-esophageal nodal lesion (Fig. 5, Image E, F&G), with marked metabolic regression of most bilateral pulmonary lesions (Fig. 5, Image H). Residual FDG-avid lesions persisted in the lingula and posterior basal segment of the left lower lobe, the larger measuring 1.7 cm (SUVmax ∼ 5.1) (Fig. 5, Image F).

**Fig. 5:**
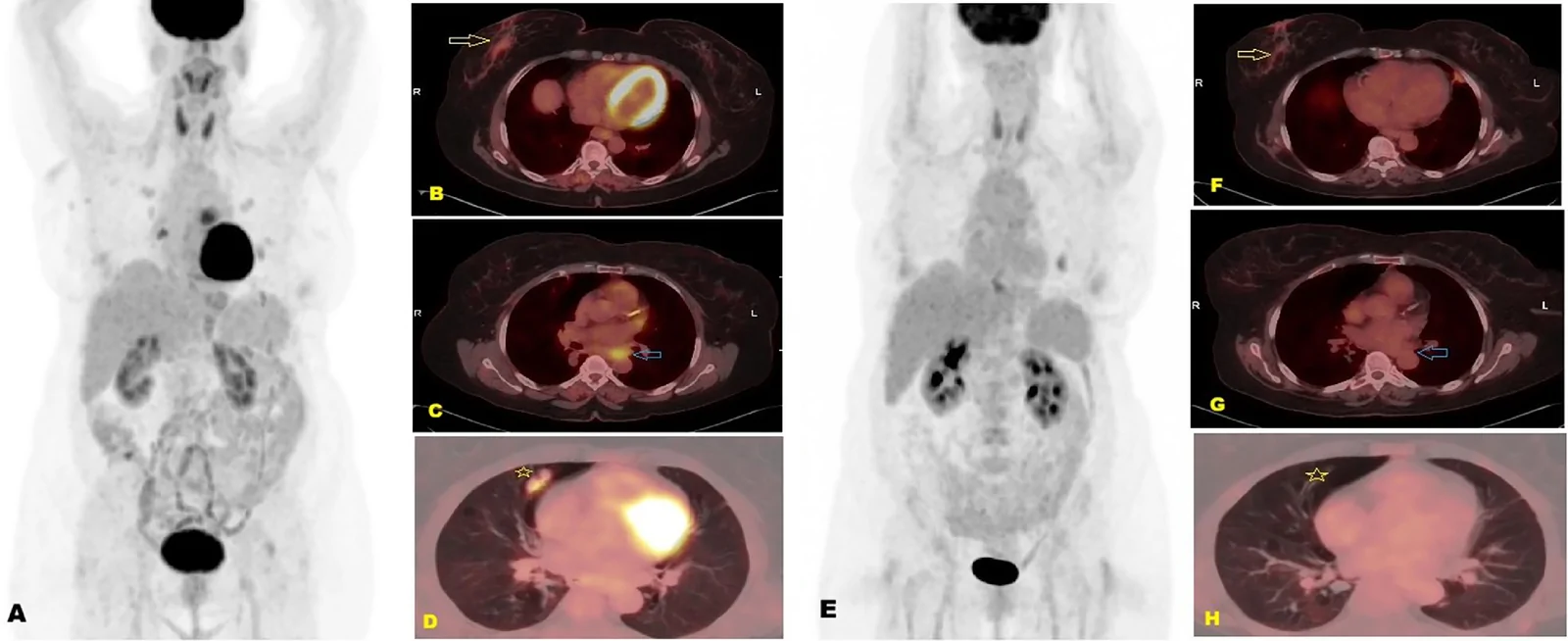
18 F-FDG PET (Maximum Intensity Projection (MIP) and fused PET-CT images): Pre-treatment 18F-FDG PET/CT images demonstrated a small FDG-avid, ill-defined right breast retro-areolar thickening measuring 4 cm (SUVmax ∼ 3) (Image A & B, Black arrow). Another FDG-avid nodal lesion was noted at the posterior mediastinal aorto-esophageal region measuring 1.4 × 2.3 cm (SUVmax ∼ 7.8) (Image A&C, Blue arrow), and bilateral pulmonary nodules with the most FDG-avid lesion in the right mid-lung zone para-cardiac region measuring 1.9 cm (SUVmax ∼ 6.8) (Image A & D, star). Follow-up 18F-FDG PET/CT performed two months later, after two cycles of V-EPOCH, showed complete metabolic resolution of the breast lesion and posterior mediastinal aorto-esophageal nodal lesion (Image E, F & G, arrows), with marked metabolic regression of most bilateral pulmonary lesions (Image H, star). Residual FDG-avid lesions persisted in the lingula and posterior basal segment of the left lower lobe, the larger measuring 1.7 cm (SUVmax ∼ 5.1) (Image F).

## Discussion

Lymphoma of the breast is a rare entity, representing approximately 0.04% to 1% of all breast malignancies, with almost exclusive female incidence. It may be present as a primary extra-nodal disease termed primary breast lymphoma or as a manifestation of secondary systemic disease. The low incidence is believed to be due to the paucity of lymphoid tissue in the breast. Histologically, diffuse large B-cell lymphoma (DLBCL) is the predominant subtype, followed by mucosa-associated lymphoid tissue (MALT) lymphoma [4,10].

Breast lymphomas often have nonspecific clinical symptoms and imaging features that mimic primary breast carcinoma. Patients usually present with a rapidly growing palpable painless breast mass, which is often solitary; however, bilateral involvement has been documented. The literature reports that 40% of masses are in the upper outer quadrant of the breast, whereas 14% are found in the retro-areolar region. Other local symptoms, such as skin changes, nipple retraction, and nipple discharge, as well as systemic B symptoms, including fever, weight loss, and night sweats, that typically present in disseminated lymphomas, are usually absent in breast lymphomas [10,11].

Mammographically, breast lymphomas typically present as a hyperdense, solitary mass with irregular or indistinct margins. Calcifications are usually absent, and the surrounding parenchymal architecture is often preserved. Similarly, sonographic findings of breast lymphoma are non-characteristic. Masses may be hypoechoic, isoechoic, or of mixed echotexture. An echogenic onion peel-like rim posterior acoustic enhancement may be present in pseudocystic masses. However, in cases of infiltrative or diffuse involvement, mammographic and sonographic interpretation may be challenging and can result in false-negative findings [6,7]. Additionally, ultrasound can aid in differentiating lymphoma from inflammatory processes in symptomatic patients, as both can present with overlapping histopathological features. Inflammatory masses typically appear as complex cysts with internal, freely mobile echoes, which are uncommon in lymphomas [6]. In the present case, the suspicious imaging findings, in conjunction with the patient’s history of PBL, raised a high suspicion for malignancy and possible recurrence, necessitating histopathological confirmation.

The imaging differential diagnosis of a breast mass in a patient with a history of plasmablastic lymphoma includes primary breast carcinoma, primary breast lymphoma, inflammatory breast carcinoma, and other malignant breast lesions. Primary breast carcinoma is commonly present as an irregular, spiculated, or indistinct mass, architectural distortion, and, in some subtypes, suspicious calcifications. In contrast, breast lymphoma is more often present as a relatively well-defined or indistinct hyperdense mass without associated calcifications or marked architectural distortion. Inflammatory breast carcinoma may demonstrate skin thickening, trabecular and stromal edema, diffuse increased density, and an underlying mass, although these findings may overlap with inflammatory or infiltrative lymphoma. Other malignant lesions may similarly demonstrate irregular margins, increased density, or associated architectural distortion, making imaging findings alone insufficient for definitive distinction [11,12].

In the present case, the lesion demonstrated an irregular, dense mass with indistinct margins and associated trabecular changes, features considered highly suspicious for malignancy and warranting a BI-RADS 5 assessment and tissue diagnosis. The patient’s history of plasmablastic lymphoma raised concern for recurrence; however, the imaging findings were non-specific and overlapped with those of primary breast carcinoma and other malignant lesions. Histopathological confirmation was therefore necessary for definitive diagnosis.

Histopathological evaluation by ultrasound-guided core needle biopsy remains essential for definitive diagnosis. Furthermore, it allows for accurate subtyping, which provides valuable prognostic information and guides appropriate management [6]. As seen in our patient, PBL is characterized by diffuse proliferation of plasmablasts or immunoblasts with frequent mitoses and anaplastic features, while immunohistochemistry typically shows positivity for CD38, CD138, MUM1, Blimp1, XBP1, and MYC, with weak or absent expressions of CD20 and PAX5 [1,2].

The management of PBL of the breast requires a multidisciplinary approach integrating oncologic, hematologic, surgical, and radiologic expertise. Initial evaluation includes comprehensive staging with PET/CT or whole-body CT to assess systemic involvement, in addition to laboratory studies such as complete blood count, lactate dehydrogenase (LDH), HIV serology, and immunophenotyping. Bone marrow biopsy is recommended for accurate staging and prognostication [1,4,7].

Despite the absence of a universally accepted standard of care, intensive combination chemotherapy continues to serve as the first-line therapeutic approach, with various regimens explored to improve outcomes. Intensified protocols such as dose-adjusted EPOCH, CODOX-M/IVAC, or hyper-CVAD have improved response rates in aggressive histology [2,8]. The choice of dose-adjusted EPOCH reflected its use in aggressive B-cell lymphomas and plasmablastic lymphoma based on available evidence and institutional practice. Incorporation of bortezomib into chemotherapy regimens may offer additional benefit, particularly in relapsed or refractory cases [1,2]. Surgical intervention is usually confined to diagnostic biopsy, given its lack of impact on survival outcomes [4,13]. Radiotherapy may be considered in cases of localized disease, residual post-chemotherapy masses, or palliative indications [5,10]. Lastly, supportive care measures, including growth factor support, infection prophylaxis, and close monitoring for treatment-related toxicity, are integral to management. At the time of the initial diagnosis, the patient underwent surgical excision followed by adjuvant radiotherapy, as the disease was localized to the maxillary sinus. However, she was managed by chemotherapy upon later recurrence due to systemic dissemination.

PBL carries a poor prognosis, with a median overall survival ranging from 6 to 19 months [1,8]. Prognostic factors influencing survival include Ann Arbor stage, HIV status, viral load, and management intensity [1,2]. Localized disease at presentation and achievement of complete remission are recognized as favorable prognostic factors [14], which may account for the prolonged remission observed in our patient.

Spontaneous regression in aggressive lymphomas represents an exceptionally rare phenomenon, with only a few cases documented in the literature. While the underlying mechanism remains uncertain, several hypotheses have been proposed, including immune stimulation secondary to infection, immune reactivation following methotrexate withdrawal, and immune reconstitution following initiation of highly active antiretroviral therapy (HAART) in HIV-infected patients [9,[14], [15], [16]]. Other proposed mechanisms include local immune activation triggered by tissue trauma from biopsy, anti-inflammatory or immunomodulatory effects, and exposure to radiation from sources other than therapeutic radiotherapy [16]. In our patient, in the absence of other medications, intercurrent disease, or identifiable systemic triggers, we hypothesize that local immune activation induced by biopsy-related tissue trauma may have contributed to the observed local regression.

The present case is notable for occurring in an immunocompetent patient without an identifiable precipitating factor. Previously described cases of apparent spontaneous regression have predominantly involved extranodal sites such as the oral cavity and maxillofacial region [9,16], whereas breast involvement is exceedingly uncommon. While some reported cases were managed with watchful waiting and subsequently showed sustained clinical and radiologic improvement, our case showed apparent localized radiologic resolution despite systemic disease, ultimately necessitating systemic therapy.

The apparent radiologic resolution of a histopathologically proven aggressive lymphoma presents a diagnostic challenge and necessitates consideration of alternative explanations rather than assuming true biological regression. Imaging findings in hematologic malignancies should be interpreted with caution, as radiologic appearances may not always accurately reflect the underlying tumor biology. Both morphologic and functional imaging provide complementary information in lymphoma and may demonstrate dynamic changes during disease assessment, reflecting differences in anatomic tumor burden and metabolic activity [17]. Consequently, apparent resolution on conventional imaging may represent a transient or localized phenomenon rather than true remission. In the present case, the initial lesion was large, well-defined, and categorized as BI-RADS 5 on mammography and ultrasound; however, given the absence of repeat biopsy, complete biological resolution cannot be confirmed histopathologically. Therefore, both morphologic and functional imaging were utilized to further evaluate the apparent local resolution and assess for systemic disease involvement.

In this case, the discordance between complete radiologic resolution of the breast lesion and the subsequent detection of systemic disease on FDG-PET highlights these limitations, underscoring the importance of integrating imaging findings with histopathological results and comprehensive systemic staging. Recognition of such atypical and dynamic presentations may enhance diagnostic awareness and help refine imaging interpretation in complex hematologic malignancies.

## Conclusion

This case represents an uncommon presentation of plasmablastic lymphoma involving the breast, which may mimic primary breast carcinoma on imaging, underscoring the importance of radiologic–pathologic correlation, comprehensive staging, and close clinical follow-up.

## Ethical approval

The authors state that informed consent has been obtained from the patient and that she has given approval for this information to be published in this case report, along with the accompanying images. All identifying information has been removed to protect patient privacy.

## Patient consent

The authors state that informed consent has been obtained from the patient and that she has given approval for this information to be published in this case report, along with the accompanying images.

## Author contributions

**Maryam Alheddi:** Clinical information collection and manuscript writing. **Najla Alomanib:** Clinical information collection and manuscript writing **Amina Mohammed:** Manuscript revision, interpretation of imaging findings. **Rabab Alkhayyat:** Interpretation of histopathological findings. **Ahmed Alsharakhat:** Clinical information collection, interpretation of histopathological findings. **Raja Eid:** Clinical information collection, manuscript revision. **Salwa Ali:** Interpretation of imaging findings, Manuscript revision.
